# Establishing a Deep End GP group: a scoping review

**DOI:** 10.3399/BJGPO.2021.0230

**Published:** 2022-08-10

**Authors:** Daniel Butler, Diarmuid O'Donovan, Jennifer Johnston, Nigel D Hart

**Affiliations:** 1 School of Medicine, Dentistry and Biomedical Sciences,, Queen’s University Belfast, Belfast, UK; 2 Northern Ireland Medical Dental Training Agency, Belfast, UK

**Keywords:** healthcare disparities, health inequities, primary health care, family practice, general practice, social deprivation

## Abstract

**Background:**

GPs working in deprived areas, where all-cause mortality rates are higher compared to less deprived areas, face unique challenges. Despite 50 years passing since Tudor Hart’s seminal ‘inverse care law’ paper, the health inequities gap remains wide. Deep End GP groups are frontline GP-led initiatives working to close this gap and improve the health and lives of those most in need.

**Aim:**

To use scoping methodology to map out the process of creating a Deep End GP group.

**Design & setting:**

A scoping review using Arksey and O’Malley’s framework.

**Method:**

MEDLINE, Embase, Web of Science, and CINAHL databases, as well as non-peer reviewed publications, were searched and articles extracted, reviewed, and analysed according to iterative inclusion criteria.

**Results:**

From an initial search number of 35 articles, 16 articles were included in the final analysis. Key steps in starting a Deep End GP group were: quantifying patients and practices in areas of deprivation; establishing GP-led objectives at an initial meeting; regular steering group meetings with close collaboration between academic and frontline general practice, as well as the wider multidisciplinary team; and adopting a local Deep End logo.

**Conclusion:**

Deep End GP groups have made advances to reduce health impacts of systemic health inequities. Starting a Deep End GP group involves a multidisciplinary approach, beginning with the identification of patients and practices in areas of highest need. The findings and key themes identified in this scoping review will guide interested parties to start the journey to do the same in their locality and to join the Deep End movement.

## How this fits in

The Deep End GP network, originating in Scotland in 2009, pools the experience and ideas of GPs in the most deprived areas. This enables advocacy, mitigates burnout, and provides practical, grassroots interventions to improve patient care in areas with the highest patient need. This scoping review mapped the process of establishing a new Deep End GP group, based on the success of existing Deep End GP groups across the world, providing a framework for other colleagues to do the same.

## Introduction

Compared to the most affluent populations, deprived populations in the UK have increased levels of multimorbidity, with disease onset 10–15 years earlier, significantly higher mortality rate,^1–3^ and an increased association with mental health morbidity.^1,4–6^ GPs working in areas of deprivation experience increased demand for GP appointments^1^ and are under increased stress,^7^ with more patients registered per GP.^1,5,6,8^ This is not a new problem, as explained in 1971 by the late Julian Tudor Hart, in his seminal ‘inverse care law’ article, which posits that those in most need of good health care are least likely to be able to access it.^9^ Despite significant efforts to address health inequities and inequalities within general practice, progress has been slow.

The Scottish Deep End Project was established in 2009. It brought together GPs working within areas of high deprivation.^10,11^ Each subsequent project serves the most deprived populations, facilitating advocacy and engagement with the public and patients to influence health policy and practice. There are currently 11 Deep End GP groups across five countries, with growing interest in the establishment of new groups.^12^ The positive impacts have been numerous, for example, the recruitment and training of younger GPs through Deep End training schemes,^13^ as well as the introduction of projects supporting GPs with protected time to integrate and align with social workers and other members of the multidisciplinary team.^11^

The COVID-19 pandemic continues to expose existing health inequities,^14^ demonstrated by COVID-19 mortality rates, which, like all-cause mortality rates, are higher in more deprived areas.^15^ The *Build Back Fairer* report^14^ called for multisectoral action; the formation and development of Deep End GP groups is one example of how general practice can mobilise its collective voice and resources to make a difference in shaping health and social care systems.

The rationale for this scoping review was to map the processes and themes involved in starting a Deep End GP group, based on the successes of existing Deep End GP groups across the world. Using this process as an evidence base, the authors plan to establish their own Deep End GP group, in their locality, as well as providing a framework for other colleagues to do the same. A scoping review was chosen over a systematic review, as the appropriate tool of data synthesis in determining the scope and coverage of literature in this area, particularly given that not all articles were empirical.^16^ Scoping reviews focus on breadth, and are regarded as the favourable methodology to systematically map the available literature and summarise the research findings.^17,18^ The process was strengthened by the use of reference scanning and grey literature searches as part of the stepwise methodology.^17,19–22^

## Method

The scoping review followed the processes and steps set by Arksey and O’Malley,^17^ and was informed by more recent publications around scoping review methodology.^19,20,22,23^

This article aimed to answer what the literature tells us about how to establish a Deep End GP group.

### Inclusion and exclusion criteria

In scoping methodology, inclusion and exclusion criteria (see Table 1) were iteratively developed based on increasing familiarity with the field of study. The main inclusion criteria were publications, including grey literature, since 2009 about Deep End GP groups across the world, in the English language.

**Table 1.** Inclusion and exclusion criteria

**Inclusion criteria**
Publications about Deep End GP groups across the world Articles published from 2009 onwards English language only articles Any empirical, discussion, or editorial articles 'Grey’ literature
**Exclusion criteria**
Publications before the Deep End Project was first formed in 2009 Articles not in the English language Blogs

### Search strategy

MEDLINE, Embase, Web of Science, and CINAHL databases were selected to locate articles on the scoping review topic. A search strategy combining key terms was developed and used in the database searches with the aid of a librarian. See Supplementary Appendix S1 for the MEDLINE search strategy.

### Study selection

Titles and abstracts of all studies and articles were screened by two independent reviewers based on relevance to the scoping review question, initially by title screening, then second level screening of abstracts, and finally full-text screening. Where differences in selection occurred, this was resolved through discussion. A third reviewer was available to resolve any disputes. Rayyan software^24^ was used to manage the study selections.

Reference lists were searched for additional relevant publications. A grey literature search followed. Each available Deep End GP group website was reviewed and if an appropriate published report was found (including online repositories) it was included. These then underwent abstract and full-text screening.

### Data extraction

A modified data extraction table, as set out by the Joanna Briggs Institute,^25^ was used for data collection and coding. Key findings related to the research question were characterised, summarised, and subsequently assigned coding (see Supplementary Box S1).

### Data analysis and presentation

Descriptive thematic analysis was performed, alongside extraction of demographic data, and charted according to Arksey and O’Malley’s framework.^17^ Data have been presented in a descriptive format that aligned with the objective of establishing future Deep End GP groups, built off the evidence and success of previous Deep End initiatives.

## Results

### Descriptive results

**Figure 1.**
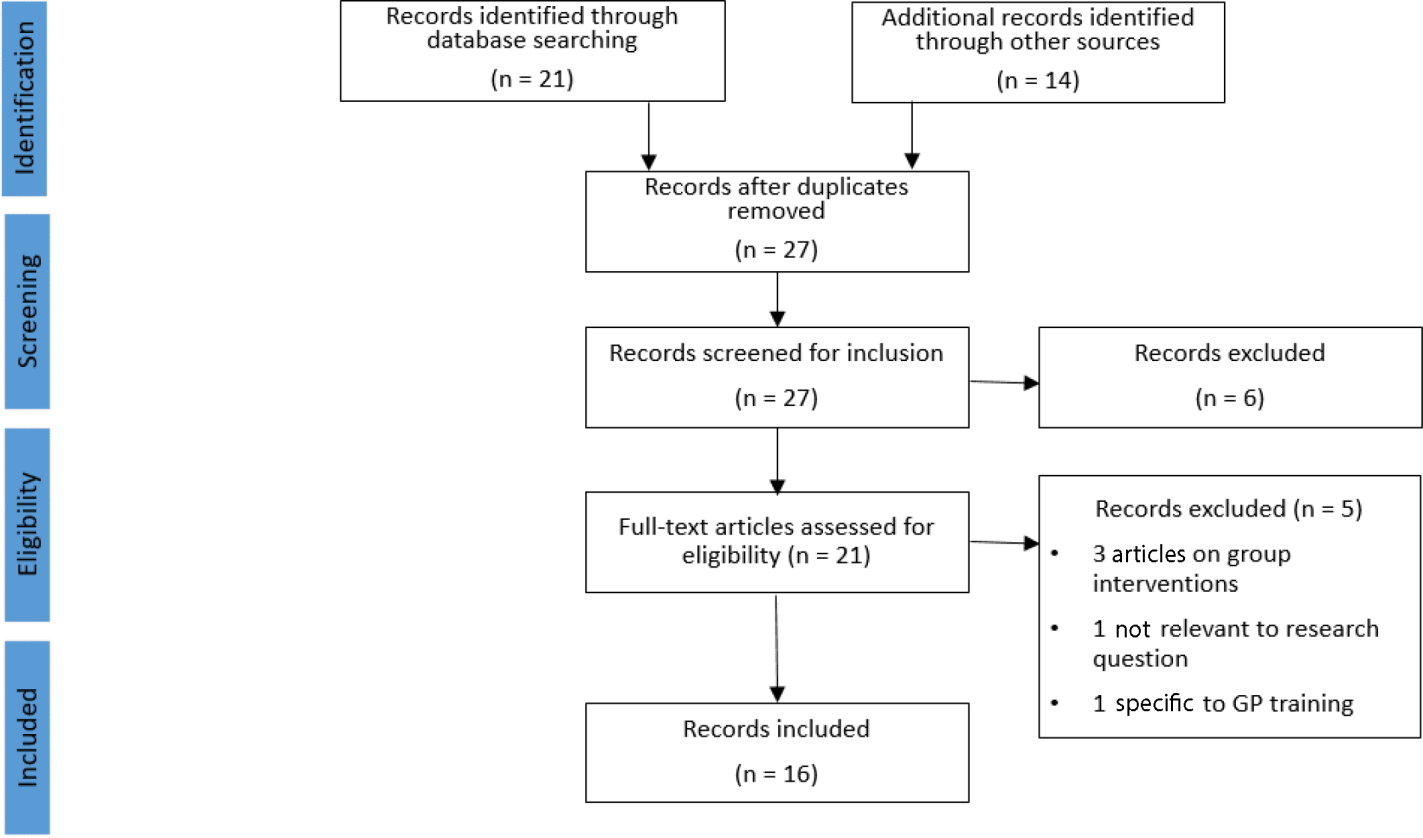
PRISMA flow diagram

A total of 16 articles were included in the review.^10,11,26–39^ Figure 1 outlines the process by which these articles were chosen.

The final selection comprised nine peer reviewed journal articles, one book publication, and six reports published outside of peer review journals.

Ten results were authored or co-authored by Graham Watt of the University of Glasgow, who was central to the instigation of the initial Scottish Deep End Project and the subsequent international Deep End GP groups. All, except one article, were published in UK journals, with the BJGP publishing seven of nine articles.

The results were published over 11 years since the first Deep End Project commenced in 2009. Peaks were seen in 2012 and 2019 coinciding with increased publication around the first Deep End work in 2011–2012 and the subsequent International Deep End bulletins.

### Thematic results

Seven overarching themes emerged when establishing a Deep End GP group, listed below.

#### 1: Quantify where the Deep End is

Quantifying areas of deprivation and the general practices that serve them was the initial step identified. Most groups (*n* = 8) used quantitative methods to identify those GPs and patient groups in areas of deprivation, mapping the Deep End.^10,11,31–38,36–38^ The most common modality used was that of the initial Scottish Deep End Project, where the Indices of Multiple Deprivation were used to rank practices with the highest proportion of patients living in 15% most deprived data zones.^10,11,34–36^

Other groups (*n* = 2) used an alternative self-identifying approach, whereby GPs self-identified as working in an area of socioeconomic deprivation.^26,30^ This was seen outside of the UK, in Australia and Ireland, due to the difficulty of accessing similar data to that used within the UK NHS.^11^ Thus, in these areas it was not possible to follow the example of the Scottish Deep End Project in quantifying practices in areas of deprivation.

A third approach also emerged when establishing a Deep End GP group, which combined both inviting practices identified in mapped areas of quantified deprivation and inviting individuals that self-identified as working in areas of deprivation.^11^ This pragmatic third option is an expansion of the original model of quantifying ‘blanket deprivation’, which allowed practices and practitioners working in areas of ‘pocket deprivation’ to self-identify and join the Deep End projects in their locality. The Scottish Deep End Project acknowledged the focus on blanket deprivation accounts for 50% of those living in deprivation,^36^ but misses the other 50% of the population served by practices that were not part of a Deep End project. This third approach minimises that exclusion.

#### 2: Host an initial meeting where participants establish the group’s future objectives

Once the Deep End had been quantified in a geographical area, all the groups had an initial meeting where the groups’ objectives were established through a process of co-design with the attending participants within each group.

The theme of participant co-design and objective setting is seen in all the groups,^11,26,29–34,36^ with the Deep End framework aiming to respect and value GPs working in the deprived communities by *‘putting them front and centre with academics and health service personnel acting as consultants in the process‘*.^27^ Specific aims or objectives were not formally drawn up until a first meeting with the GPs who work at the Deep End.

One objective commonly included by Deep End GP groups was advocacy for both patients and GPs in areas of high need.^26,29,33–39,39^

#### 3: Secure funding; desirable but not essential

The funding received when establishing the different Deep End GP groups varied. The initial Scottish Deep End Project successfully received funding to backfill 100 locum GPs, providing locum cover allowing practitioners from each of the 100 most deprived practices to be represented, as well as funding for attendance at steering group meetings.^11,31,34,36^

Deep End Yorkshire and Humber received initial funding from Health Education England Yorkshire and Humber,^33^ and later formed a research cluster that has received funding from the National Institute for Health Research Clinical Research Network.^11^ The Northeast and North Cumbria Deep End GP group secured funding from the Northeast and North Cumbria North of England Commissioning Support Unit.^37^ When establishing the Greater Manchester Deep End GP group, funding was allocated from a charitable trust, with the group sitting within the Shared Health Foundation Community Interest Company.^11^

The benefits of formal funding were seen through some groups being able to employ a project manager to help coordinate the groups, such as the Yorkshire and Humber and Northeast and North Cumbria Deep End GP groups.^37^ Securing funding has, however, not been a prerequisite to establishing a Deep End GP group. The Deep End GP groups in Canberra and Ireland have been established successfully without any major funding, with GPs attending in their own time.^26,30^ The difficulty of this model is it may exclude GPs with significant workloads, working 4–5 days a week from attending. Being more suitable for less than full-time colleagues may possibly impact the number and range of practices represented in a funded model.^35^

#### 4: Establish a smaller steering group

A common theme following an initial meeting is the formation of smaller focused working or steering groups. This has enabled the different groups to unite a variety of voices from the Deep End, empowering ongoing advocacy for general practice in the most deprived areas while following and being accountable to the agenda and objectives set out by the larger collective meetings. The strength of peer support and regular contact with peers in similar working environments is also a key strength of ongoing productive Deep End GP groups, providing common purpose, support, and motivation.^11,26,28,30,31,34,38^ In Scotland, *‘the beating heart of the Deep End Project has been its steering group, comprising of 15–20 general practitioners‘*.^11^

#### 5: Collaborate with academic general practice

A strong collaboration between academic and ‘frontline’ general practice emerged. The initial Scottish Deep End Project sought to bring together GPs serving the most deprived areas, enabling the practitioners’ voices to be united.^26^ Subsequently, at least half (five out of 10) of the groups were facilitated by academic GP colleagues based in universities.^11,30,34^

The connection with academic general practice supports evidence gathering, inspired by issues and experiences raised by frontline Deep End colleagues, as well as academically rigorous evaluations of interventions and pilots that have been undertaken.^11,32,33^

#### 6: Decide on membership eligibility

The results showed a mixed approach to those recruited and included within the different Deep End GP groups, with all groups being initially established and led by GPs. Most groups started exclusively with GPs, as seen in Scotland,^10,35,36^ Ireland,^30^ North and West London,^32^ and Canberra.^26^ This, however, has evolved to include other allied primary care colleagues within primary care and general practice teams, including frontline GPs, nurses, practice managers, researchers, educators, medical students, and public health colleagues.^11,33,38^

#### 7: Adopt a Deep End logo

**Figure 2.**
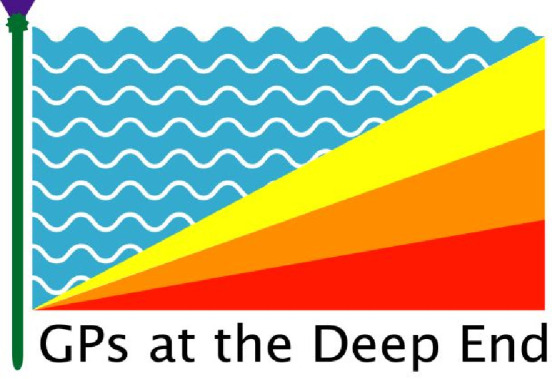
Deep End Scotland logo^34^

Deep End GP groups have adopted a similar logo initially inspired by the Scottish Deep End Project (see Figure 2).^11,26,34^ It demonstrates, in pictorial form, the realities of life and work at the Deep End, with it being harder for patients and practitioners to keep their metaphorical heads above water as deprivation and associated need deepens. It is a uniting aspect of all the individual groups as they merge to become a collective voice of the international network for general practice at the Deep End of deprivation.

## Discussion

### Summary

The scoping methodology on how to establish a Deep End GP group mapped the first step of quantifying and defining the Deep End of general practice for a geographical area. Ideally, this method used a quantitative approach, then invited representatives of practices in the most deprived areas. However, where this was not possible, GPs successfully established groups by inviting self-identified Deep End colleagues.

Early participant co-design and objective setting, led by the practitioners who work in high need, high deprivation areas, was key to establishing a successful Deep End GP group. This not only gathered momentum but ultimately helped to achieve the aims it specifically set out. Deep End GP groups built with an academic general practice partnership have benefited in developing a strong evidence base of the issues faced and in reviewing the successes of piloted solutions. In turn, this has strengthened the advocacy and voice of practitioners working at the Deep End. Funding and allied health care involvement are beneficial, but not a prerequisite to a successful and impactful group.

For colleagues interested in embarking on this journey, visiting the Scottish Deep End Project website is essential reading. In particular, reviewing the work of the different groups within the International Deep End reports. Colleagues wanting to express their interest in starting a Deep End GP group are encouraged to use the contact details found on the Scottish Deep End Project website.^40^

### Strengths and limitations

This scoping review was timely in the shadow of 50 years of the ‘inverse care law’^9^ and as healthcare systems begin to reflect on the impact of the COVID-19 pandemic on society, especially the high mortality rate among people living in the most socioeconomically deprived areas.^14,15^ As measures to address health inequalities and inequities are considered, the initiative and impact of Deep End GP groups uniting general practice will be increasingly important.

The low number of articles in this search limited the extent and impact outside of a Deep End framework or initiative. It may be that other initiatives or projects that bring GPs in the most deprived areas together have not been included. The review also focused on formation and establishment of a Deep End GP group rather than the impact and successes of individual groups.

Scoping methodology is different from systemic review methodology. In focusing on breadth, it allows a map of existing literature to be created and gaps identified. It also allows for the inclusion of multiple types of data without a quality assessment. While no formal synthesis akin to meta-analysis is part of a scoping review, some degree of synthesis was undertaken in descriptive thematic analysis. A systemic review may sometimes be an appropriate follow-up to a scoping study, but the authors felt this was less valuable given the high number of non-empirical articles.

### Comparison with existing literature

There has been a plethora of innovation and practice changing interventions published by Deep End GP groups since 2009. As of December 2021, 11 Deep End GP groups have been established,^12^ these groups are modelled on previous well-established groups. This review brought together and synthesised the approaches taken in establishing a Deep End GP group, aiming to provide a framework for other interested colleagues across the UK and further afield looking for a pragmatic approach to address the health inequalities they see in daily practice.

### Implications for practice

This study set out to establish how to create a GP network based on the Deep End model, as a tool for colleagues to improve general practice and the health outcomes in areas of high need. The key steps identified start with quantifying patients and practices in areas of deprivation; establishing locally specific, GP-inspired objectives at an initial meeting; introducing regular steering groups meetings, with close collaboration between academic and frontline general practice, as well as the wider multidisciplinary team; and finally adopting a local Deep End logo.
